# How multifunctional can a single fiber be?

**DOI:** 10.1093/nsr/nwag133

**Published:** 2026-03-03

**Authors:** Zhixun Wang, Lei Wei

**Affiliations:** Department of Electrical and Electronic Engineering, The Hong Kong Polytechnic University, China; School of Electrical and Electronic Engineering, Nanyang Technological University, Singapore

The world’s first electrical battery was the voltaic pile, an aqueous battery that uses a water-based electrolyte, invented by Alessandro Volta in 1800 [1]. Although later lithium batteries have dominated the commercial market due to their high capacity and other superior electrochemical performance, aqueous batteries still attract significant attention as promising candidates for safer energy storage to avoid the potential fire hazards of lithium batteries. Commonly, aqueous batteries are solely thought of as energy storage devices. In a recent paper, however, the Qichong Zhang’s group and collaborators revealed that, when designed with a fiber-shaped architecture, aqueous batteries can do much more—they not only store energy but also rectify and enable neuromorphic computing mimicking brain connections.

A smart textile with system-level attributes requires multiple modules, such as power sources, power management circuits, sensors, data processing units, and user interfaces [2]. Integration of these modules into planar devices is usually achieved through a system-on-a-chip (SoC) solution. For fiber-based devices, integrating these modules poses significant challenges due to difficulties in building soft, conformal, and reliable connections [3]. Zhang and co-workers have demonstrated an all-in-one approach to assembling batteries, diodes, and artificial synapses, seamlessly integrating three functions into a single fiber by harnessing the unique dual-ion transport mechanism of aqueous batteries [4].

**Figure 1.**
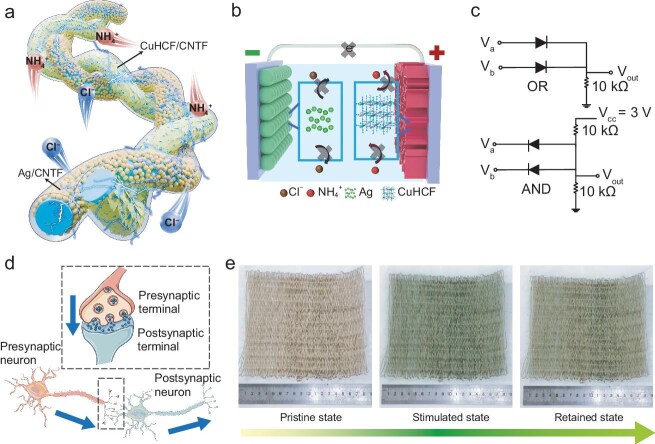
(a) Schematic of the fiber-shaped aqueous dual-ion batteries (FADIBs). (b) Under reverse bias, the suppression of Faradaic reactions minimizes the current and leads to the unidirectional current flow in FADIBs. (c) Equivalent circuit diagram of OR and AND gates constructed by FADIBs. (d) Schematic of neuronal signal transmission. (e) Modulating electrochromic fabric via FADIB-based artificial synapse-type fiber circuit. Adapted with permission from Ref. [4].

The fiber-shaped aqueous dual-ion batteries (FADIBs) consist of a CuHCF/CNTF cathode, an Ag/CNTF anode, and an NH_4_Cl/PVA gel electrolyte (Fig. 1a). The key innovation lies in regulating both Faradaic and non-Faradaic processes to enable voltage-dependent ion migration. When functioning as a battery, the device delivers a high capacity of 70.5 mAh cm^−3^ and an energy density of 51.5 mWh cm^−3^ as NH_4_^+^ ions reversibly intercalate into the CuHCF cathode while Cl^−^ ions participate in redox reactions at the Ag anode. The authors also proposed exploiting

asymmetric ion transport routes to achieve ionic diode functionality. Under forward bias, both Faradaic reactions at the cathode and the anode contribute to the current, whereas under reverse bias, the suppression of Faradaic reactions minimizes the current (Fig. 1b). This asymmetry leads to a rectification ratio of up to 10^9^. The rectification behavior was demonstrated in fiber-based logic gates (Fig. 1c) and in a fiber-based rectifier for triboelectric nanogenerators. Furthermore, Zhang and co-workers realized that ion migration and redistribution emulate biological synaptic plasticity (Fig. 1d). Under pulsed stimuli, the FADIBs reproduce excitatory and inhibitory postsynaptic currents and spike-rate-dependent plasticity, with ultra-low energy consumption of only 7.5 fJ per synaptic event. Zhang and co-workers combined FADIBs with electrochromic fibers to create a soft, flexible smart device. The device has a fully textile appearance without any rigid components and is capable of self-powering, self-regulating, and color-changing (Fig. 1e). The FADIBs provide power and are also used as artificial synapse-type management circuits to control electrochromic fibers.

In sum, Zhang and co-workers show that aqueous batteries, through the rational design of ion-transport mechanisms, can transcend their original energy-storage function and serve as fiber-shaped flexible multifunctional devices with a power source, rectifier, and data-processing unit all within a single thread. This all-in-one approach offers a compelling strategy for the development of future textiles, where our clothes do not just cover us, but also think and respond.
